# A new hybrid method with data-characteristic-driven analysis for artificial intelligence and robotics index return forecasting

**DOI:** 10.1186/s40854-023-00483-5

**Published:** 2023-04-10

**Authors:** Yue-Jun Zhang, Han Zhang, Rangan Gupta

**Affiliations:** 1 Business School, Hunan University, Changsha, 410082 China; 2 Center for Resource and Environmental Management, Hunan University, Changsha, 410082 China; 3 Department of Economics, University of Pretoria, Private Bag X20, Hatfield, 0028 South Africa

**Keywords:** Artificial Intelligence and Robotics index return forecasting, PSO-LSSVM model, GARCH model, Decomposition and integration model, Combination model, Q43, G15, E37

## Abstract

Forecasting returns for the Artificial Intelligence and Robotics Index is of great significance for financial market stability, and the development of the artificial intelligence industry. To provide investors with a more reliable reference in terms of artificial intelligence index investment, this paper selects the NASDAQ CTA Artificial Intelligence and Robotics (AIRO) Index as the research target, and proposes innovative hybrid methods to forecast returns by considering its multiple structural characteristics. Specifically, this paper uses the ensemble empirical mode decomposition (EEMD) method and the modified iterative cumulative sum of squares (ICSS) algorithm to decompose the index returns and identify the structural breakpoints. Furthermore, it combines the least-square support vector machine approach with the particle swarm optimization method (PSO-LSSVM) and the generalized autoregressive conditional heteroskedasticity (GARCH) type models to construct innovative hybrid forecasting methods. On the one hand, the empirical results indicate that the AIRO index returns have complex structural characteristics, and present time-varying and nonlinear characteristics with high complexity and mutability; on the other hand, the newly proposed hybrid forecasting method (i.e., the EEMD-PSO-LSSVM-ICSS-GARCH models) which considers these complex structural characteristics, can yield the optimal forecasting performance for the AIRO index returns.

## Introduction

Artificial Intelligence and Robotics are key technologies of the Fourth Industrial Revolution which are rapidly changing how people live and work. Since the onset of the COVID-19 pandemic, the WHO-advised social distancing has led to a more virtual existence, which may accelerate the development of Artificial Intelligence (AI) and Robotics technologies further. During the past decade, AI technologies have been hailed by many academics and practitioners as revolutionary and game-changing in the business world, a sphere in which the AI and robotics activities have significantly increased (Felten et al. 2018; Furman and Seamans 2019; Gruetzemacher et al. 2021; Mikalef and Gupta 2021).

Meanwhile, AI and robotics stocks have also attracted wide investor attention, and the investment in AI has grown rapidly (Bughin et al. 2017). According to the AI Index 2021 annual report, despite the pandemic, 2020 still saw a 9.3% increase in private AI investment from 2019, a higher percentage jump than in 2019 (5.7%). Furthermore, the statistical data also shows that the United States remains the leading destination for private investment, with over USD 23.6 billion in funding in 2020, followed by China (USD 9.9 billion) and the United Kingdom (USD 1.9 billion) (Zhang et al. 2021a; b). Therefore, AI and robotics technology companies are exerting a growing influence on the financial market, representing an interesting investment option for portfolio diversification. It is evident that the growth in AI investment trend is consistent, and seizing this smart investment boom has become an important question. Therefore, relevant investors must first choose a reliable index reflecting the investment opportunities associated with AI technology. Further, they need to make an accurate analysis and prediction of index returns, which could help them dynamically grasp the evolution rule for the entire range of this industry, and enable them to reasonably develop the optimal portfolio strategy (Zhang and Wang 2019; Zhang et al. 2020; Ghosh et al. 2022).

At present, the indices related to AI and robotics mainly include the Nasdaq CTA Artificial Intelligence and Robotics Index (NQROBO Index), the Global Robotics and Automation Index (ROBO Index), and the Indxx Global X Robotics & Artificial Intelligence Index (IBOTZ Index). The Nasdaq CTA Artificial Intelligence and Robotics Index (hereafter referred to as the AIRO index) is designed to track the performance of companies engaged in the AI and robotics segment of the technological, industrial, medical, and other economic sectors. Therefore, this index is the most important among the three major indices since it can comprehensively reflect the overall stock price change and the associated development of the AI industry. Based on its price data, it can be established that from December 19, 2017 to July 23, 2021, the cumulative return rate of the AIRO index reached 84.84%, and the annualized return rate was 33.92%. The movement in this index is closely tied to other financial assets (Le et al. 2021; Tiwari et al. 2021). Thus, it is essential to accurately forecast the AIRO index returns which can provide a reference for investors to select suitable index funds and investment tools, and to help them target the investment opportunities of the growing AI and robotics industries.

However, the literature on AIRO index returns forecasting is relatively scarce, and most of the research focuses on AI progress forecasting, and the application of AI technology in forecasting tasks (Chang et al. 2018; Mascio et al. 2021). In particular, research on the nonlinear and time-varying characteristics of this index is scarce, and needs to be supplemented. Currently, the commonly used financial time-series forecasting models can be classified into traditional econometric models and machine-learning methods; both possess advantages and disadvantages when used in forecasting. For example, the traditional econometric models are usually effective in capturing the linear and time-varying components, but they cannot fully capture nonlinear components and have several requirements for data stability (Hung 2011; Lin et al. 2011; Zhang et al. 2015). However, the machine-learning methods are suitable for predicting nonstationary, nonlinear time series because of their flexible nonlinear function-fitting capabilities and less-restrictive assumptions, but their forecasting performance is easily affected by data size and parameter settings (Wang et al. 2005; Psaradellis and Sermpinis 2016). The literature further shows that single models, characterizing a specific feature of the data, usually cannot identify all states and correlations in complex time series (Khashei and Bijari 2011). Consequently, it affects the forecasting accuracy, since they are unable to extract the inherent dynamics. Given these limitations, the hybrid models gradually emerged in the financial time-series prediction literature (Zhang and Zhang 2018; Li et al. 2021; Xiao et al. 2021). Against this background, the issues relevant to the AIRO index returns involve the following: the data characteristics this index exhibits, and designing a reliable prediction method that accurately explores the intrinsic structural characteristics of AIRO index returns.

Hence, this paper focuses on the structural characteristics of the AIRO index and attempts to combine the econometric models and machine learning methods to develop a hybrid forecasting approach, given the complexity of the data-generating process of the AIRO index. Specifically, this paper first employs the ensemble empirical mode decomposition (EEMD) method to decompose the AIRO index return series into a series of intrinsic mode functions (IMFs) and the residual term. Further, it uses a modified version of the iterative cumulative sum of squares algorithm (ICSS) to identify the structural breakpoints. Second, different models (namely, the least-square support vector machine approach with the particle swarm optimization method (PSO-LSSVM) and the generalized autoregressive conditional heteroskedasticity (GARCH) type models) are developed to forecast the IMFs and the residual term, respectively, with the sum of forecasted values for all components being the final forecasting results of the decomposition and integration models. Finally, this paper employs two methods to combine the econometric and machine learning models, and constructs innovative hybrid forecasting models that consider the complexity of the data-generating process of the AIRO index.

The contribution of this paper involves three main aspects: (1) Previous research has primarily focused on the common comprehensive indices in the financial market, such as the S&P500 index; see Rapach and Zhou (2021) for a detailed discussion of the literature associated with international stock market forecasting. However, these indices cannot reflect and predict the development of the AI industry. This paper focuses on, conducting an in-depth analysis and forecasting of the Nasdaq CTA Artificial Intelligence and Robotics Index to provide additional insights and implications for traders. This can enable them to make informed investment decisions, while operating in different time horizons. (2) Previous studies have usually employed the single forecasting model; however, it cannot systematically capture the inherent structural characteristics of overall index returns (Rapach and Zhou 2013; Tiwari et al. 2016). Hence, this paper attempts to employ the EEMD and the modified ICSS algorithms to mine the structural features in the AIRO index returns. The AIRO index returns not only have linear and nonlinear characteristics, but also high complexity and mutability, which provide the basis and guidance for constructing the relevant measurement and mathematical models used for the AIRO index. (3) Based on the complex inherent characteristics of the AIRO index, this paper is unique in exploring appropriate forecasting models from multiple perspectives for the AIRO index returns. These empirical findings provide fresh evidence for investors and portfolio managers concerning hedging and diversification benefits in the era of the Fourth Industrial Revolution.

The empirical results imply that given the data characteristics, the hybrid model (i.e., EEMD-PSO-LSSVM-ICSS-GARCH) can overcome the limitations of a single model and effectively depict the time-varying, nonlinear, complex and mutable characteristics of the AIRO index returns. Thus, this model achieves superior forecasting performance for the AIRO index returns, providing investors with a reliable reference for portfolio selection and asset management. Moreover, this paper uses the forecasting results of the new hybrid model to construct different portfolio strategies, finding that it can improve the forecasting performance of the single models, but also increase their economic value.

The remainder of this paper is organized as follows: “Literature review” section reviews the relevant literature. “Methods” section briefly describes the models, “Data descriptions” section presents the data set, “Results and discussions” section discusses the empirical results, and “Conclusions and future work” section offers the concluding remarks.

## Literature review

In the recent past, the adoption and use of AI and robotics technologies in several industries has increased significantly (Acemoglu et al. 2018; Felten et al. 2018; Furman and Seamans 2019; Graetz and Michaels 2018; Webster and Ivanov 2020). For instance, Furman and Seamans (2019) showed that while the worldwide shipments of robots rose by approximately 150% between 2010 and 2016, and the share of jobs demanding AI skills was nearly five times higher in 2016 than in 2013. Acemoglu and Restrepo (2018) indicated that while AI and robotics can help to increase productivity growth, these new technologies will render labor redundant. Webster and Ivanov (2020) indicated that AI and robotics were all-pervading in various aspects of the economy, including manufacturing, trading in financial markets, chatbots in customer relationship management, and so on. Enholm et al. (2022) discussed the impact of AI, on the evolution of organizations, leading to competitive performance, and identified several implications of AI on the process and the firm.

Currently, many scholars choose the AIRO index, which can well represent the performance of technology-intensive companies in the AI and robotics fields, to depict the development of the AI industry. For instance, Tiwari et al. (2021) chose this index and employed the time-varying Markov-switching copula models to provide evidence of a time-varying Markov tail-dependence structure and dynamics between AI and carbon prices. Huynh et al. (2020) used this index to explore the role of AI and robotics stocks, green bonds, and Bitcoin in portfolio diversification, and proved that the portfolios of these assets exhibited heavy-tail dependence. Demiralay et al. (2021) investigated the interdependence between AI and robotics stocks and traditional and alternative assets. They identified the weak (strong) co-movements between AI and other investments in shorter (longer) investment horizons.

However, these studies focus on the correlation between the AIRO index and other financial assets, but lack the systematic research on index return forecasting. Most of the forecasting research focuses on AI progress forecasting, or the application of AI technology in the forecasting field (Xiao and Ke 2021). For instance, Chang et al. (2018) implemented the social network (SN) technique to examine a corporation’s competitive edge. They fed business relationship and financial information into an AI-based technique to construct a forecasting model. Gruetzemacher et al. (2021) described the development of a research agenda for forecasting the progress of AI. It utilized the Delphi technique to elicit and aggregate experts’ opinions on which questions and methods to prioritize.

Thus, it can be seen that previous research has used this index widely, indicating that it can well track the performance of technology-intensive companies active in the AI and robotics sector. Yet, research on return forecasting for this index has yielded no progress. However, many scholars point out that in the financial market, accurately predicting the return sequence of financial assets is one of the most challenging tasks. It is also a crucial aspect of investors’ ability to formulate portfolio strategies. This is important in pricing assets, and evaluating portfolio performance.

To date, many scholars have focused on stock index return forecasting, and machine-learning models as well as traditional econometric models have been widely used to do so (Zhang and Wang 2019; Zhang et al. 2020; Ghosh et al. 2022; Sebastio and Godinho 2021). For example, Giovannellia et al. (2021) extracted the information contained in a high number of macroeconomic predictors using large dimensional factor models to forecast the S&P 500 index return, and their results showed that the Generalized Dynamic Factor Model can help predict stock returns. Mascio et al. (2021) assessed the performance of three forecasting models to predict the one-month-ahead S&P 500 Index return (the sentiment index model) using a combined kitchen-sink forecasting model and a LASSO model. The results showed that the LASSO model outperformed the other ones. Salisu and Vo (2020) used a historical average-based model to evaluate the relevance of health-news trends in predicting stock returns during the COVID-19 period. Their results revealed that the model incorporating the health-news index outperformed the benchmark model. Thus, the great theoretical and practical significance to predict the index return in the financial market is clear. In this regard, both the traditional measurement, and the machine-learning models have attracted considerable attention.

The relevant literature indicates that the single models, characterizing a specific feature of the data, usually cannot identify all states and correlations in complex time series (Khashei and Bijari 2011). However, some studies indicate that both machine-learning and traditional econometric models possess their own advantages and disadvantages in the process of forecasting (Zhang et al. 2015; Wang et al. 2005; Psaradellis and Sermpinis 2016). Hybrid models gradually start to draw attention in forecasting research. For instance, Yu et al. (2008) proposed the “decomposition-integration” hybrid models, and their results showed that hybrid models always possess better forecasting ability. Bildirici and Ersin (2013) combined the multilayer perceptron model with the new Smooth Transition Autoregressive model and the generalized autoregressive conditional heteroskedasticity (GARCH) model, which introduced the fractional integration and asymmetric property (LSTAR-LST-GARCH-MLP) model. This proved that the hybrid framework can capture the volatility clustering, asymmetry, and nonlinearity characteristics of petrol prices. Rapach et al. (2010) indicated that this combination of models can improve the prediction performance by synthesizing the feature-capturing capability of individual models. Zhang et al. (2021a; b) developed an innovative ensemble deep-learning model with dynamic error correction and multi-objective ensemble pruning to address time-series forecasting tasks. The superior forecasting performance of the proposed model was verified using time-series data (i.e., PM2.5 concentration, wind speed, and electricity price).

Overall, the research on stock return forecasting is already quite extensive, and hybrid models become widespread because they can combine the strengths of different models. What remains unsolved in the literature is return forecasting for the AIRO index. Most related research has focused on the correlation of the AI industry with other industries and the application of AI technology in the forecasting field. However, it would be beneficial to design a reliable forecasting method considering the complexity of the data-generating process of the AIRO index, which could help investors develop optimal stock investment portfolios and hedge investment risk. Therefore, this paper attempts to combine the econometric models and machine learning methods to depict the complex structural characteristics of the AIRO index returns, based on previous research on the subject. It then constructs a hybrid forecasting approach to obtain optimal forecasting performance.

## Methods

### The EEMD method

The EEMD method (Wu and Huang 2009) is selected to decompose the complex original signal into components with different characteristics while maintaining the nonstationary and nonlinear features of the original time-series data for this study on decomposing the AIRO index returns series. The main steps of the decomposition are as follows:

1. Add a white noise series $o^{i} (t)$ with a given amplitude (i.e., 0.1) to the AIRO Index returns series $x(t)$, and the new series $x^{i} (t)$ is as follows:
  1 $$x^{i} (t){ = }x(t) + o^{i} (t)$$
2. Decompose the time series $x^{i} (t)$ into *n* IMFs $c_{j}^{i} (t)$ (*j* = 1, 2,..., n) and a residual term $r^{i} (t)$ using the EMD method, and the results are as follows:
  2 $$x^{i} (t) = \sum\limits_{j = 1}^{n} {c_{j}^{i} (t) + r^{i} (t)}$$
  where $c_{j}^{i} (t)$ is the *j*th IMF in the *i*th trial.
3. Repeat steps (1) and (2) for *M* times with different white noise each time, and obtain the corresponding IMF components of the decomposition.
4. Calculate the average of the corresponding IMFs of *M* trials for the final IMFs, as follows:
  3 $$c_{j} (t) = \frac{1}{M}c_{j}^{i} (t)$$

Once the EEMD completes, the original time series can be expressed as a linear combination of IMFs and the residual term as follows:

4 $$x(t) = \sum\limits_{j = 1}^{n} {c_{j}^{{}} (t) + r(t)}$$

where $c_{j}^{{}} (t)$ (t = 1, 2,…, T) is the *j*th IMF obtained by using the EEMD method at time *t*, $r(t)$ is the final residual term, and *n* is the number of IMFs.

### The PSO–LSSVM method

#### The LSSVM method

To describe the nonlinear characteristics of the AIRO index returns better, we single out the LSSVM model, which is a typical method in machine-learning (Suykens and Vandewalle 1999), and is particularly suitable for modeling small-size samples and nonlinear problems. The specific description of the model is as follows.

Given a set of samples, $\{y_{t} ,x_{t} \} _{{t = {1}}}^{T}$, ${\mathbf{x}}_{t}$ is the input vector, and ${\mathbf{y}}_{t}$ is the output variable. Then the decision function can be defined as:

5 $$y(x) = {\mathbf{w}}^{T} \Gamma (x) + c_{bias}$$

where *w* is the weight vector, $\Gamma (x)$ represents the nonlinear function used to map the input space to a high-dimensional feature space, and $c_{bias}$ is the bias term.

The objective function of the LSSVM model is:

6 $$\begin{aligned} & \min \left( {\frac{1}{2}\left\| {\mathbf{w}} \right\|^{2} + \frac{{c_{reg} }}{2}\sum\limits_{t = 1}^{{T^{{({\text{tr}})}} }} {\sigma_{t}^{2} } } \right) \\ & s.t.\quad y_{t} = {\mathbf{w}}^{T} \Gamma (x_{t} ) + \zeta_{t}^{(tr)} + c_{bias} ,\;t = 1,2, \ldots ,T^{(tr)} \\ \end{aligned}$$

where $c_{reg}$ is the regularization constant, and $\sigma_{t}$ denotes the training error.

Next, the final outcome of the LSSVM method based on the Kuhn-Tucker conditions can be described as:

7 $$y(x) = \sum\limits_{t = 1}^{{T^{(tr)} }} {\lambda_{t} K(x,x_{t} )} + c_{bias}$$

where $K(x,x_{t} )$ denotes the kernel function. We apply the radial basis function (RBF) with a width of $\omega$ (Keerthi and Lin 2003), which can be expressed as:

8 $$K(x,\;x_{t} ) = \exp \left( { - 0.5\left\| {x - x_{t} } \right\|^{2} /\omega^{2} } \right).$$

#### The PSO method

The PSO method is a computational technique that uses a set of particles, representing potential solutions to a problem (Eberhart and Kennedy 1995). Each particle can be defined as a potential solution to the problem in a *d*-dimensional search space. Let $U_{i} = (u_{i1} ,u_{i2} , \ldots ,u_{id} )$ be the current position of particle *i*, $V_{i} = (v_{i1} ,v_{i2} , \ldots ,v_{id} )$ be the current velocity, $P_{i} = (p_{i1} ,p_{i2} , \ldots ,p_{id} )$ be the previous position, and $P_{g} = (p_{g1} ,p_{g2} , \ldots ,p_{gd} )$ be the best position among all particles, then the best positions of particle *i* is shown as:

9 $$v_{id}^{k + 1} = wv_{id}^{k} + c_{1} r_{1} [p_{id}^{{}} - u_{id}^{k} ] + c_{2} r_{2} [p_{gd}^{{}} - u_{id}^{k} ]$$

10 $$u_{id}^{k + 1} = u_{id}^{k} + v_{id}$$

where $v_{i}^{k}$ and $u_{i}^{k}$ are the current velocity and position of particle *i*, respectively; $w$ is the inertia weight; $c_{1}$ and $c_{2}$ are acceleration coefficients; and $r_{1}$ and $r_{2}$ are two independently, uniformly distributed, random variables with the range [0, 1].

#### The PSO-LSSVM method

Due to the parameters $\omega$ and $c_{reg}$ having a significant impact on forecasting accuracy, we employ the PSO method to obtain the optimal parameters (Eberhart and Kennedy 1995); hence, the main steps of the PSO-LSSVM approach can be described as follows:

*Step 1* Take the parameters ($\omega$,$c_{reg}$) as swarms, and initialize a population of particles with random positions and velocities.

*Step 2* Evaluate the fitness of each particle based on the following fitness function:${\text{Fitness}} = [\frac{1}{N}\sum\limits_{i = 1}^{20} {(\hat{y}_{i} - y_{i}^{2} )} ]^{1/2}$, where $y_{i}$ and $\hat{y}_{i}$ represent the actual and forecast AIRO Index returns, respectively.

*Step 3* Update the previous and global best fitness values according to the fitness evaluation results.

*Step 4* Update the velocity and position values for each particle until the stop conditions are satisfied based on Eq. (9) and Eq. (10) (i.e., the number of iterations reaches a maximum of 100, or the optimal parameters satisfy the accuracy requirement, i.e., the value of fitness is less than 0.001).

### The GARCH model

To capture the time-varying character of the movements for the AIRO index returns, we employ the GARCH model proposed by Bollerslev (1986), which is the most commonly used econometric model for analyzing the volatility of returns in financial markets.^1^ The model is defined as follows:

$$r_{t} = \delta r_{t - 1} + u_{t}$$

11 $$u_{t} = \varepsilon_{t} \sqrt {h_{t} }$$

$$h_{t} = \alpha_{0} + \alpha u_{t - 1}^{2} + \beta h_{t - 1}$$

where $u_{t}$ represents the residual series, and $h_{t}$ is the conditional variance. When $t = 1, \ldots ,n$, $\varepsilon_{t}$ ~ N(0, 1), the model should satisfy $\alpha_{0} > 0$, $\alpha \ge 0$, $\beta \ge 0$ and $\alpha + \beta < 1$.

To depict the structural changes in the AIRO index returns, this paper combines the structural breakpoints with GARCH (1,1); the variance equation is shown in Eq. (12).

12 $$h_{t} = \omega + d_{1} D_{1} + \cdots + d_{n} D_{n} + \alpha u_{t - 1}^{2} + \beta h_{t - 1}$$

where $D_{1} , \ldots ,D_{n}$ are dummy variables that are determined according to the structural breakpoints identified by the modified ICSS algorithms (Ewing and Malik 2017).

### The hybrid method for forecasting AIRO index returns

The hybrid method is capable of modeling both nonlinearity and time variations, which indicates that it may possess better forecasting ability in terms of the AIRO index returns. In this circumstance, we attempt to construct a hybrid model based on the decomposition and integration, and model combination methods. The procedures can be described as follows:

1. The EEMD method is used to decompose the original AIRO index return series to obtain the IMFs and the residual term.
2. We normalize the decompose IMFs components and residual term, and appropriately select training and testing samples. Then, the single models above (i.e., the GARCH-type and PSO-LSSVM models) are used to forecast the IMF components and the residual term, respectively.
3. The forecasting results of each IMF component and residual term are superimposed to obtain the final forecasting results of the decomposition and integration models (i.e., the EEMD-GARCH-type and EEMD-PSO-LSSVM models).
4. The following two methods are used to obtain the hybrid predictions:
5. The GARCH-type models are built to predict high-frequency IMFs with time-varying characteristics, whereas the PSO-LSSVM model predicts low-frequency IMFs and residual terms with nonlinear characteristics. Next, the final forecasting results of the new hybrid model are obtained by superimposing the above forecasts, i.e., the EEMD-PSO-LSSVM-GARCH(A) and the EEMD-PSO-LSSVM-ICSS-GARCH(A) models.
6. We combine the forecasting results of the EEMD-GARCH-type and the EEMD-PSO-LSSVM in Step (3) using the mean combination approach, and the new hybrid models, i.e., the EEMD-PSO-LSSVM-GARCH(B) and EEMD-PSO-LSSVM-ICSS-GARCH(B),^2^ are used to obtain the final forecasting results.

### The evaluation criteria for forecasting performance

In accordance with Hansen and Lunde (2005), we apply two widely used statistical loss functions, i.e., the Mean Square Error (MSE) and the Mean Absolute Error (MAE)—to evaluate the out-of-sample forecasting performance for the AIRO index returns, which are defined as Eqs. (13)–(14):

13 $$MSE = \frac{1}{T - N}\sum\limits_{t = N + 1}^{T} {(\hat{h}_{t} - h_{t} )^{2} }$$

14 $$MAE = \frac{1}{T - N}\sum\limits_{t = N + 1}^{T} {\left| {\hat{h}_{t} - h_{t} } \right|}$$

where *h*_*t*_ represents the actual return, whereas $\hat{h}_{t}$ represents the forecasted return; *T* and *N* represent the number of full- and in-sample observations, respectively, and *T* − *N* is the number of out-of-sample observations.

Meanwhile, we use the Model Confidence Set (MCS) method proposed by Hansen et al. (2011) to judge whether the models used have a statistically significant difference in forecasting performance. In particular, the range statistic is chosen in this study, i.e., $T_{R} = \mathop {\max }\nolimits_{a,b \in M} \frac{{\left| {\overline{g}_{ab,t} } \right|}}{{\sqrt {{\text{var}} (g_{ab,t} )} }}$, where $\overline{g}_{ab,t}$ denotes the relative performance variable of model $a$ and $b$. The range statistic and its corresponding *p*-value are obtained using a bootstrap procedure. Following Hansen et al. (2011) and Wang et al. (2016), we consider a confidence level of 90%, which means that a model with the MCS *p* value larger than 0.1 will be included in the MCS.

## Data descriptions

**Fig. 1:**
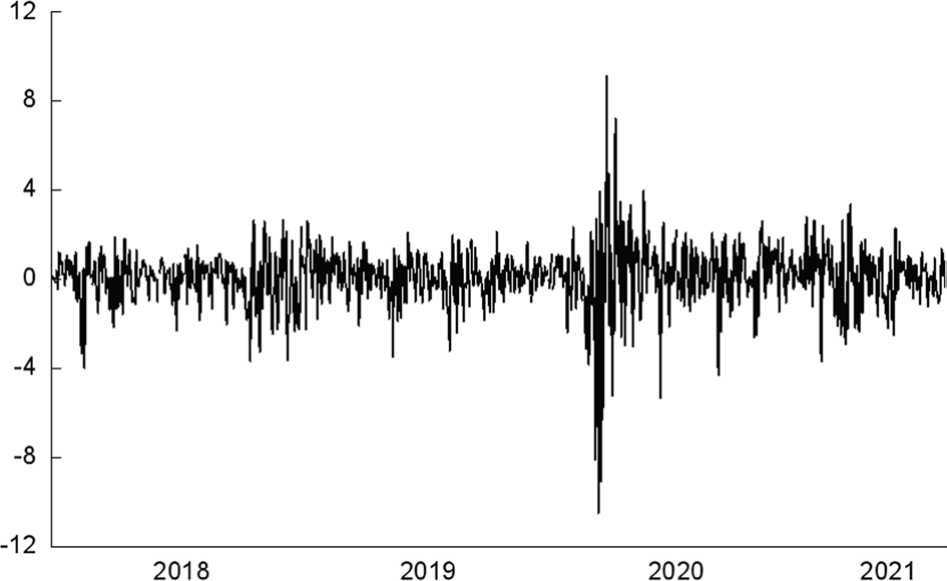
The AIRO index log-returns

Following Huynh et al. (2020), this study chooses the daily AIRO index price data from the NASDAQ market as the research focus, with the data obtained from *Bloomberg*.^3^ The AIRO index reflects the innovation level of the market and the performance of the AI industry in the era of the Fourth Industrial Revolution (Tiwari et al. 2021). The full sample ranges from 12/19/2017 to 07/26/2021, and the specific sample periods for the training and testing samples are 12/19/2017 to 10/13/2020 and 10/14/2020 to 07/26/2021, respectively. The AIRO index returns are calculated as *r*_*t*_ = 100 × [*log*(*p*_*t*_) − *log*(*p*_*t*−1_)], where *p*_*t*_ indicates the AIRO index price at time *t*. The daily AIRO index log returns are shown in Fig. 1.

Table 1 presents the descriptive statistics of the AIRO index returns. It can be observed that the AIRO index returns series has negative skewness and positive excess kurtosis, suggesting the presence of a leptokurtic and fat-tailed distribution. Moreover, the Jarque–Bera test results indicate that the null hypothesis of a normal distribution is rejected at the 1% significance level. The Ljung–Box Q-statistics for the squared returns also reject the null hypothesis of no autocorrelation up to the 10th order at the 1% significance level, which indicates the existence of autocorrelation in the volatility of the AIRO index returns. Table 1 also presents the results of the unit root tests. Specifically, the results of the Augmented Dickey-Fuller (ADF—Dickey and Fuller 1981) test and the Phillips–Perron (PP—Phillips and Perron 1988) test reject the null hypothesis of a unit root at the 1% significance level, indicating that the AIRO index returns are stationary over the sample period.

**Table 1:** Descriptive statistics of the log-returns of the AIRO index

	AIRO index returns		AIRO index returns	
Mean	0.0672	*Q*(10)	91.354 (0.0000)	
SD	1.3775	*Q*^2^(10)	619.02 (0.0000)	
Skew	− 0.9783	ADF	− 8.8190 (0.0000)	
Kurtosis	13.2993	PP	− 29.2280 (0.0000)	
*J–B*	4180.96 (0.0000)			

The *p* values are reported in parentheses. SD represents the standard deviation. J–B is Jarque–-Bera test statistic, with the null hypothesis of normal distribution. *Q*(10) and *Q*^2^(10) denote the Ljung–Box *Q*-statistics of the returns and squared returns series for up to 10th order serial autocorrelation. ADF and PP are the statistics of the augmented Dickey–Fuller and Phillips–Perron unit root tests, respectively, based on lags determined by the Akaike Information Criterion (AIC)

## Results and discussions

### The EEMD decomposition results

**Fig. 2:**
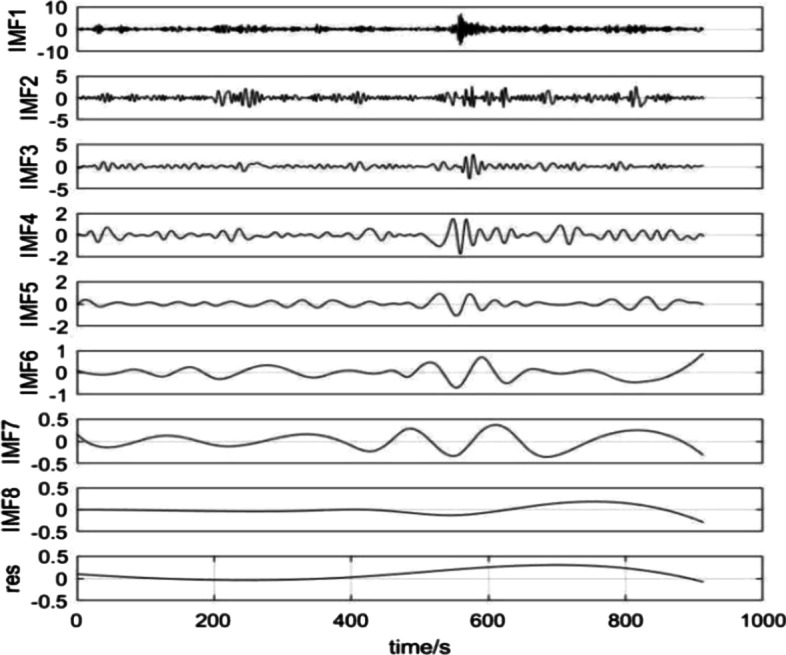
EEMD decomposition of log-returns for the AIRO index

Based on this discussion regarding methods, we obtain the EEMD decomposition result for the AIRO index returns in Fig. 2. First, the original AIRO index returns series is decomposed into eight independent intrinsic mode functions and one residual term, defined as sub-series in the following section, using the EEMD method. As depicted in Fig. 2, the IMFs obtained by the EEMD algorithm are irregular, which is caused by the nonlinear and noise components of the AIRO index returns. In addition, the frequency of the eight IMF components and the residual term are arranged from high to low, which shows the diversity of the AIRO index returns in terms of the frequency and multi-scale characteristics. Furthermore, it shows that the AI industry may be affected by various factors, including the strong uncertainty regarding the industrial chain, and the development of AI technology. Specifically, the average period of the IMF1-IMF5 is relatively short, which is the high-frequency component of the original AIRO index returns series, and reflects the impact of short-term irregular events on the AI industry. The GARCH-type models are used to forecast these sub-series.

The average period of IMF6-IMF8 is relatively long, indicating the impact of major events in the field of artificial intelligence, while the PSO-LSSVM model is applied to forecast these sub-series. Moreover, the residual term has declined slowly since September 2019, which reflects that under the impact of economic fundamentals—industrial structure adjustment, macro policy, and so on—the AIRO index returns have declined since September 2019. Investors can capture the AI industry development via this long-term trend, and grasp the investment risks and returns, which enable them to look for new AI industry investment opportunities.

### Structural breaks test in AI and robotics market

This paper uses the modified version of Inclan and Tiao’s (1994) iterated cumulative sum of squares (ICSS) algorithm to identify six structural breakpoints in the AIRO index returns series, and divides the sample period into seven intervals accordingly. The results are presented in Table 2. It is worth noting that the structural break in February 2020 was closely related to the COVID-19 pandemic, and “OpenAI Five” beat humans. Accenture invested in China and focused on an artificial intelligence layout for the first time in August 2018, causing a breakpoint in the AIRO index returns. The disease diagnosis based on AI technology made a great breakthrough for the breakpoint in February 2018, indicating that AI can revolutionize the diagnosis and management of diseases through a large amount of data analysis and classification. These results confirm that structural breaks in the AIRO index returns tend to occur due to emergencies or external shocks.

**Table 2:** Structural breakpoints in the AIRO index returns, as detected by the ICSS algorithm

No	Break point	Time period	Variance
		2017/12/19–2018/2/1	
1	2018/2/1	2018/2/2–2018/4/9	1.2705
2	2018/4/9	2018/4/10–2018/8/3	0.7396
3	2018/8/3	2018/8/4–2019/1/25	1.6029
4	2019/1/25	2019/1/26–2020/2/21	0.8942
5	2020/2/21	2020/2/22–2020/5/13	3.4373
6	2020/5/13	2020/5/14–2021/7/26	1.2177

This paper employs the modified version of the ICSS algorithm to detect the structural breakpoints. The specific sample periods are from 2017/12/19 to 2021/7/26

### Forecasting results of AIRO index returns

We examine the forecasting performance of all competitive models, in order to find the optimal forecasting model for AIRO index returns based on the randomness, periodicity, and trend of this series. First, we consider a single forecasting model without the data decomposition method, i.e., the traditional econometric model (GARCH-type models) and the machine-learning framework (PSO-LSSVM method). Second, we employ the GARCH-type and the PSO-LSSVM models to forecast all the sub-series, respectively, and obtain the forecasts from integrated-decomposed models (i.e., the EEMD-GARCH-type and EEMD-PSO-LSSVM models). Third, we use two methods to combine the EEMD-GARCH-type and EEMD-PSO-LSSVM models and derive forecasts from the final new hybrid models (i.e., EEMD-PSO-LSSVM-ICSS-GARCH (A) and (B) models). Finally, we calculate the loss function values and corresponding MCS test of 1-day ahead forecasting results for the daily log-returns of the AIRO index to evaluate the predictive abilities of the different models. The forecasting results are presented in Table 3. From this table, we identify the following findings.

1. The MSE and MAE values indicate that the forecasting performances of the GARCH and the PSO-LSSVM models are not significantly different. The hybrid PSO-LSSVM and GARCH models perform better than the two single models, i.e., the EEMD-PSO-LSSVM-GARCH(A) and (B) models. The results show that the PSO-LSSVM model can better capture the nonlinear characteristics of the AIRO index returns, and has the advantages of nonlinear mapping, self-learning, and self-organization. On the contrary, the GARCH model has the advantage of capturing the time-varying and volatility-clustering characteristics of the AIRO index returns. The results also suggest that the hybrid models can consider the linear and nonlinear characteristics of the AIRO index returns, and combine the advantages of the PSO-LSSVM and GARCH models. This helps in obtaining superior forecasting performance compared to the single model.
2. The models that consider structural changes can achieve even better predictive performance. As shown in Table 3, the ICSS-GARCH and EEMD-ICSS-GARCH models have lower forecasting losses than the models without structural changes. The improved predictive performance is particularly evident for the new mixed models that consider structural breakpoints. Huynh et al. (2020) point out that as these firms are participants in the not yet mature AI market, AI stocks may react significantly to changes in other asset markets. Therefore, incorporating structural breakpoints can better capture the response of the AI market to emergencies, leading to the models making more accurate predictions.
3. Compared to the single models, the decomposition-integration models usually perform better in their ability to forecast the AIRO index returns. Specifically, as shown in Table 3, the values of MSE and MAE always indicate that the forecasting results of the EEMD-GARCH-type and EEMD-PSO-LSSVM models are significantly better than those of the corresponding models that do not apply the EEMD algorithm. Moreover, the decomposition-integration models are always included in MCS under more criteria than single models with a confidence level of 90%. This result shows that the single model is greatly affected by the characteristics of the AI industry and the data itself, such that its prediction ability is weaker than that of the decomposition-integration models. Thus, the EEMD method can account for the periodicity, randomness, and trend characteristics of the AIRO index returns. This method effectively decomposes the original sequence into simple modes to obtain stable IMFs components and the residual term, thereby improving the forecasting accuracy. Moreover, the results can help investors mine the forecasting information of the AI industry index more comprehensively and measure the investment risks more reasonably.

**Table 3:** 1-Day ahead forecasting results for the daily log-returns of the AIRO index

Models	MSE	MAE
GARCH	1.3760	0.8885
ICSS-GARCH	1.3584	0.8837
PSO-LSSVM	1.3530	0.8807
EEMD-GARCH	0.6745	0.6277
EEMD-ICSS-GARCH	0.6661	0.6294
EEMD-PSO-LSSVM	0.6738	0.6375
EEMD-PSO-LSSVM-GARCH(A)	0.6433	0.6089
EEMD-PSO-LSSVM-GARCH(B)	0.6423	0.6087
EEMD-PSO-LSSVM-ICSS-GARCH(A)	0.6330	0.5932
EEMD-PSO-LSSVM-ICSS-GARCH(B)	**0.6280**	**0.5894**

The numbers in the table refer to the values of the two loss functions. The bold numbers indicate that the corresponding models have the lowest forecasting losses. The underlined numbers indicate that the corresponding models are excluded in the MCS under the corresponding criteria

The final new hybrid models involving the structural characteristics mentioned above always result in superior forecasting. As can be seen from Table 3, the values for the two loss functions involving the two final hybrid models are significantly lower than those of the other models. Additionally, the MCS test results also show that compared with other models, the final hybrid models are included in the MCS under more criteria. This indicates that the hybrid models can consider the linear and nonlinear, complexity, and mutability characteristics of the AIRO index returns, thereby obtaining superior forecasting performance compared with other models. Particularly, the values of MSE and MAE are significantly reduced with the hybrid models compared with the others, and the final mean combination model (i.e., the EEMD-PSO-LSSVM-ICSS-GARCH(B) models) usually performs the best out of all the models considered. The results show that the AI industry is affected by multiple factors. Thus, the AIRO index returns present multiple characteristics, and the final hybrid model combined with the EEMD method and the modified ICSS algorithm can help investors effectively capture the complexity of the industry index. Hence, they can change their investment strategy to adapt to the changing financial market, and obtain steady income streams under different investment risks.

### Economic significance

During the past decade, global investors have paid wide attention to the stocks of AI and robotics companies to reap the potential investment benefits. In order to judge whether the new models can help AI market investors gain higher investment benefits, this paper further uses the mean–variance investment strategy to investigate the economic values of AIRO index returns forecasting models from an asset allocation perspective (Ferreira and Santa-Clara 2011; Xing and Zhang 2022). The main forms are as follows:

Assuming that a mean–variance investor optimally allocates between the AIRO index and risk-free bills based on the various return forecasts, the utility *U*_*t*_ of portfolio strategy *P* can be defined as follows:

15 $$\begin{aligned} U_{t} (R_{t}^{P} ) & = E_{t} (R_{t}^{P} ) - 0.5\gamma Var_{t} (R_{t}^{P} ) \\ & = \omega_{t} (r_{t}^{e} + r_{t}^{f} ) + (1 - \omega_{t} )r_{t}^{f} - 0.5\gamma \omega_{t}^{2} \sigma_{t}^{2} \\ \end{aligned}$$

where $E_{t} ( \bullet )$ and $Var_{t} ( \bullet )$ represent the conditional mean and variance of the portfolio return $R_{t}^{P}$ at time *t*, respectively. $r_{t}^{e}$ and $\sigma_{t}^{2}$ are the AIRO index excess return and volatility on day *t*, respectively. $r_{t}^{f}$ is the risk-free rate, $\gamma$ is the investor’s coefficient of relative risk aversion, and $\omega_{t}$ is the portfolio weight. By maximizing the objective function, we can obtain the optimal portfolio weight $\omega_{t}^{ * }$, given by

16 $$\omega_{t}^{ * } { = }\frac{1}{\gamma }\left( {\frac{{\hat{r}_{t + 1}^{e} }}{{\hat{\sigma }_{t + 1}^{2} }}} \right)$$

where $\hat{r}_{{t{ + }1}}^{e}$ and $\hat{\sigma }_{{t{ + }1}}^{2}$ are the out-of-sample forecasting value of excess returns and volatility, respectively. Specifically, we apply the forecasting models above to ensure the value of $\hat{r}_{{t{ + }1}}^{e}$, and use the prevailing historical average to forecast $\hat{\sigma }_{{t{ + }1}}^{2}$. We restrict the value of $\omega_{t}^{ * }$ between 0 and 1.5 because of the short-sale constraint. Then, we compute the portfolio return $R_{t + 1}^{P}$ as:

17 $$R_{t + 1}^{P} = \omega_{t}^{ * } (r_{t + 1}^{e} + r_{t + 1}^{f} ) + (1 - \omega_{t}^{ * } )r_{t + 1}^{f}$$

The mean–variance investor who allocates assets using Eq. (17) can realize the certainty equivalent return (CER), defined in Eq. (18):

18 $${\text{CER}} = \hat{\mu }_{p} - \frac{\gamma }{2}\hat{\sigma }_{p}^{2}$$

where $\hat{\mu }_{p}^{{}}$ and $\hat{\sigma }_{p}^{2}$ are the sample mean and variance, respectively, of the portfolio return over the out-of-sample evaluation period.

Using the method above, this paper proposes different portfolio strategies under different optimal weights, which are determined according to the return forecasts above. Further, we calculate the average portfolio return (R) and certainty equivalent return (CER). It should be noted that the higher values of CER usually mean a greater economic value of the corresponding portfolio strategy, i.e., the economic significance of the corresponding model is more positive in practical applications. The test results are shown in Table 4. Hence, we have the following findings.

**Table 4:** Portfolio performance based on the various AIRO index return forecasting models

Model	$\gamma$ = 3		$\gamma$ = 6	
	CER	R	CER	R
GARCH	5.8206	8.1122	5.1192	8.0959
ICSS-GARCH	5.9958	8.3265	5.3127	8.5046
PSO-LSSVM	6.0763	8.5270	5.0061	8.3902
EEMD-GARCH	6.0251	9.8281	5.4408	9.1478
EEMD-ICSS-GARCH	6.4976	10.1235	6.9680	9.6550
EEMD-PSO-LSSVM	6.2975	9.4479	5.6500	10.1256
EEMD-PSO-LSSVM-GARCH(A)	7.2490	11.1524	7.0463	10.8509
EEMD-PSO-LSSVM-GARCH(B)	7.5180	10.1599	7.0558	11.7524
EEMD-PSO-LSSVM-ICSS-GARCH(A)	7.8384	12.4724	7.1233	10.3698
EEMD-PSO-LSSVM-ICSS-GARCH(B)	8.1890	12.1558	7.5918	11.8649

The underlined numbers indicate that the corresponding models have the highest R or CER among all models

First, the R and CER values of the portfolio strategy are relatively higher for forecasting models that consider structural changes, and the decomposition-integration models also have better economic value. As shown in Table 4, the values for the models combining the EEMD methods (the EEMD-GARCH and EEMD-PSO-LSSVM models) are mostly better than those for single models (the GARCH and PSO-LSSVM models). Similarly, the economic value of the models combining structural breakpoints is largely better than the others, which enables investors to consider this factor when developing portfolio strategies to achieve better returns.

Second, the new hybrid model exhibits the best economic performance. As shown in Table 4, the R and CER values of the final hybrid models are always the highest (i.e., EEMD-PSO-LSSVM-ICSS-GARCH(A) and (B)), which suggests that these can capture the complex characteristics of the AIRO index simultaneously, and thus yield the best economic value.

### Robustness checks

#### Different data frequencies

The diversity of investor behavior normally results in data exhibiting various characteristics at different frequencies. Therefore, to examine whether and how the central empirical results change over different data frequencies, and to further judge whether the hybrid model is suitable for investors with different trading horizons, this paper replaces the daily data with weekly data, while the full sample ranges remain unchanged. Specifically, we use weekly data to re-estimate the models, with the specific periods of training and testing samples being 12/19/2017–08/23/2020 and 08/24/2020–07/26/2021, respectively, given the full sample of 12/19/2017–07/26/2021.

As Table 5 shows, the MSE and MAE values of the EEMD-GARCH and EEMD-PSO-LSSVM models are lower than those of the GARCH and PSO-LSSVM models without the EEMD method. This shows that the EEMD method can effectively decompose the AIRO index return series with noise, allowing us to obtain more accurate data for the subsequent prediction process. Hence, the decomposition-integrated forecasting models proved to be better than the single models at a weekly frequency as well.

**Table 5:** 1-Week ahead forecasting results for weekly log-returns of the AIRO index

Model	MSE	MAE
GARCH	5.6166	1.8659
ICSS-GARCH	5.6836	1.8563
PSO-LSSVM	5.8967	1.9826
EEMD-GARCH	2.7728	1.2973
EEMD-ICSS-GARCH	2.7684	1.2948
EEMD-PSO-LSSVM	2.7412	1.3188
EEMD-PSO-LSSVM-GARCH(A)	2.7085	1.2966
EEMD-PSO-LSSVM-GARCH(B)	2.6995	1.2869
EEMD-PSO-LSSVM-ICSS-GARCH(A)	2.6832	1.2957
EEMD-PSO-LSSVM-ICSS-GARCH(B)	2.6856	1.2746

The numbers in the table refer to the values of the two loss functions. The underlined numbers indicate that the corresponding models have the lowest forecasting losses

Furthermore, the final hybrid models also yield superior forecasting performance compared to the other models. Specifically, the MSE and MAE values of the final hybrid models (A and B models) are significantly lower. This shows that investors with both, long and short trading horizons, can consider the new hybrid model to capture the complex industry characteristics and forecast the AIRO index returns with more accuracy. These findings have important implications for investors and policymakers in terms of portfolio diversification, risk management, asset allocation, and price regulation. Overall, our results are robust across high and low frequency data.

#### Different sample periods

Some uncertainties may affect the central results presented till now. To determine whether different sample periods can affect our findings, we select a new sample period of 07/26/2018–07/26/2021 to re-estimate the models, and the corresponding in and out-of-sample periods are chosen to be 07/26/2018–11/25/2020 and 11/26/2020–07/26/2021, respectively. The results of the 1-day ahead forecasting using this new setup are presented in Table 6. By comparing the results from the two loss functions reveals that the forecasting results of the decomposition-integration models are superior to those of the single model. In addition, compared with other models, the two new final hybrid models continue to achieve better forecasting performance, and the mean combination model (B model) performs the best among all the models. In summary, the central results are also robust to different sample periods.

**Table 6:** 1-Day ahead forecasting results for daily log-returns of the AIRO index under alternative sample periods

Model	MSE	MAE
GARCH	1.1642	0.8452
ICSS-GARCH	1.0946	0.8384
PSO-LSSVM	1.2654	0.8807
EEMD-GARCH	0.6614	0.6239
EEMD-ICSS-GARCH	0.6593	0.6183
EEMD-PSO-LSSVM	0.6674	0.6146
EEMD-PSO-LSSVM-GARCH(A)	0.5855	0.5935
EEMD-PSO-LSSVM-GARCH(B)	0.5592	0.5902
EEMD-PSO-LSSVM-ICSS-GARCH(A)	0.5686	0.5975
EEMD-PSO-LSSVM-ICSS-GARCH(B)	0.5474	0.5893

The numbers in the table refer to the values of the two loss functions. The underlined numbers indicate that the corresponding models have the lowest forecasting losses

#### Different artificial intelligence index

To prove the superiority and robustness of the final hybrid model, this paper further chooses other Artificial Intelligence and Robotics indices as research objects to depict the changes in that specific industry. Specifically, this paper selects the NYSE FactSet Global Robotics and Artificial Intelligence Index (NYFSRAI), which can track equity performance in robotics and artificial intelligence. The Robotics area mainly includes companies referring to robotics integrated applications, development, manufacturing, and the devices involved in high-speed, high-precision, and automation etc. The AI area mainly includes companies involved in AI development, programming, and software and hardware implementation. The loss function values of each model are listed in Table 7, and the main results are discussed below.

**Table 7:** 1-Day ahead forecasting results for daily log-returns of the NYSERAI index

Model	MSE	MAE
GARCH	1.6261	0.9682
ICSS-GARCH	1.5903	0.9632
PSO-LSSVM	1.6339	0.9712
EEMD-GARCH	0.9568	0.7547
EEMD-ICSS-GARCH	0.9034	0.9283
EEMD-PSO-LSSVM	0.8421	0.6611
EEMD-PSO-LSSVM-GARCH(A)	0.8410	0.6601
EEMD-PSO-LSSVM-GARCH(B)	0.7961	0.6693
EEMD-PSO-LSSVM-ICSS-GARCH(A)	0.6930	0.6354
EEMD-PSO-LSSVM-ICSS-GARCH(B)	0.7083	0.6277

The numbers in the table refer to the values of the two loss functions. The underlined numbers indicate that the corresponding models have the lowest forecasting losses

On the one hand, the decomposition-integration model still has superior forecasting ability compared to single models for the NYSERAI index. As Table 7 shows, the loss function values of the EEMD-PSO-LSSVM and EEMD-GARCH models are smaller than those of the PSO-LSSVM and GARCH models. This shows that the EEMD algorithm can effectively decompose the NYSERAI index return series containing noise, and obtain the stable IMFs and the residual term to provide more suitable data for the subsequent forecasting process. On the other hand, the final hybrid models still have the best forecasting performance for the NYSERAI index. Specifically, the values of MSE and MAE in Table 7 show that the loss function values of EEMD-PSO-LSSVM-ICSS-GARCH (A) and (B) decrease significantly.

#### Different benchmarking models

To prove the superiority of the final hybrid model in this paper, we further compare the forecasting accuracy of the hybrid model with some recognized benchmarking models, such as neural network models according to Fang et al. (2020). The daily data is trained with the same interval as above, to obtain objective comparison results. Specifically, we set the full sample range from 12/19/2017 to 07/26/2021, and the specific sample periods for the training and testing samples are 12/19/2017 to 10/13/2020 and 10/14/2020 to 07/26/2021. The 1-day ahead forecasting results of the different benchmarking models are listed in Table 8. A comparison with Table 3 shows that the final hybrid models outperform the benchmarking models. For example, the MSE values of the EEMD-PSO-LSSVM-ICSS-GARCH (A) and (B) are 0.6330 and 0.6280, respectively, whereas those of the DNN, LSTM, PSO-BP, and GA-ELM are 1.7882, 1.6924, 1.7660, and 1.6252, respectively. This further proves the robustness of the central conclusion.

**Table 8:** 1-Day ahead forecasting results for daily log-returns of the AIRO index of different benchmarking models

Model	MSE	MAE
DNN	1.7882	1.0067
LSTM	1.6924	0.9711
PSO-BP	1.7660	1.0082
GA-ELM	1.6252	0.9368
EEMD-PSO-LSSVM-ICSS-GARCH(A)	0.6330	0.5932
EEMD-PSO-LSSVM-ICSS-GARCH(B)	0.6280	0.5894

The numbers in the table refer to the values of the two loss functions. The underlined numbers indicate that the corresponding models have the lowest forecasting losses

#### Transaction cost

The transaction cost may make a difference in the performance of the portfolio (Guidolin and Pedio 2021). Therefore, to judge whether the results of the economic value test are robust, this study assumes that there are 30 basis points of transaction cost when trading assets. The new results are presented in Table 9. We find that the CER and R values based on the final hybrid models are relatively higher than those of the other models. This shows that when the transaction cost is considered, we can still prove the robustness of the economic significance results.

**Table 9:** Portfolio performance based on the various AIRO index return forecasting models including transaction cost

Model	$\gamma$ = 3		$\gamma$ = 6	
	CER	R	CER	R
GARCH	6.0206	9.3271	5.8226	8.5831
ICSS-GARCH	6.1165	9.3118	5.9793	9.2696
PSO-LSSVM	6.1306	9.4493	6.2501	9.3859
EEMD-GARCH	6.9194	10.4653	6.6018	9.7862
EEMD-ICSS-GARCH	7.0716	10.1235	6.9803	10.0194
EEMD-PSO-LSSVM	6.5572	10.2998	6.3590	10.2606
EEMD-PSO-LSSVM-GARCH(A)	8.1190	11.0244	7.2438	11.0990
EEMD-PSO-LSSVM-GARCH(B)	8.0180	11.3953	7.1586	11.4274
EEMD-PSO-LSSVM-ICSS-GARCH(A)	8.9445	13.0896	7.6390	12.9952
EEMD-PSO-LSSVM-ICSS-GARCH(B)	9.1566	12.5948	7.5882	11.9839

The underlined numbers indicate that the corresponding models have the highest R or CER among all models

## Conclusions and future work

To accelerate the development of the AI industry globally, relevant industries must examine this rapidly changing AI market and make innovative investments. Therefore, from the investor perspective, it is crucial to understand the Artificial Intelligence index. In order to mine the intrinsic structural characteristics of the AIRO index returns deeply and comprehensively, and to judge which type of model can better predict the AIRO index returns, this paper is the first one attempting to combine machine-learning techniques with traditional econometric models based on the “decomposition-integration” and “model combination” methods for the AIRO index returns forecasting. Specifically, the EEMD method and modified ICSS algorithm are used to analyze the data characteristics, and the basic single models in this paper include the PSO-LSSVM and GARCH models. The main conclusions are drawn as follows.

First, the EEMD decomposition and integration method significantly improves the forecasting performance of the single models of the AIRO index returns. This is mainly because the EEMD method can obtain a more stable and simple mode, giving full consideration to the periodicity, randomness, and trend characteristics of the AIRO index returns. Consequently, more accurate forecasting results are obtained, driven by the features of the data. In addition, the result is valid regardless of whether it is for the PSO-LSSVM or GARCH models. This further proves the applicability of the decomposition and integration method to the AIRO index returns.

Second, regardless of whether we use daily or weekly data and different sample periods, the forecasting performance of the GARCH and PSO-LSSVM models is not significantly different. Additionally, the hybrid model (i.e., the EEMD-PSO-LSSVM-GARCH model), which combines these frameworks, can markedly improve the forecasting performance of the single models. This result shows that the traditional econometric model is suitable for describing the time-varying characteristics in the AIRO index returns; the machine-learning model can better capture the nonlinear characteristics; and the hybrid model can effectively combine their advantages.

Third, the AIRO index returns exhibit complex structural characteristics. Specifically, it not only presents time-varying and nonlinear characteristics, but also possesses high complexity and mutability. In a context where most AI market participants are not mature, the structural change caused by an external shock plays a critical role in predicting the AIRO index return. Additionally, the final hybrid model, which further considers structural change (i.e., the EEMD-PSO-LSSVM-ICSS-GARCH model), can comprehensively capture the complex characteristics of the AIRO index returns, and yield the best forecasting performance and economic value.

These conclusions have clear theoretical and practical implications. On the one hand, we perfect the research framework in the field of Artificial Intelligence. Previous research has focused on the correlation between the AI industry and other industries or the application of AI technology in the forecasting field. We further focus on the AIRO Index, and conduct an in-depth analysis and forecasting, thereby perfecting the research framework in the field of Artificial Intelligence; On the other hand, based on the essential characteristics and pattern characteristics exhibited by the AIRO index returns, we propose the optimal forecasting model (i.e., EEMD-PSO-LSSVM-ICSS-GARCH model). The final forecasting model can overcome the limitations of a single model, which further expands the relevant forecasting theory.

The conclusions in this paper also have several practical implications for both policymakers and investors interested in portfolio diversification. First, the financial market participants can utilize the EEMD-PSO-LSSVM-ICSS-GARCH model to capture and mine more of the data characteristics of the AIRO index returns. This can help them make more accurate forecasting decisions, which can provide an important reference for them to target investment opportunities, prevent risks, and reap benefits in the AI industry. Second, forecasting of the AIRO index returns can help the policymakers understand the future changes in the AI stock market in a timely way. This can result in the formulation of effective policies to maintain the financial market and social stability, involving financial market risk management, option pricing etc.

In the future, there is still much interesting work to be explored regarding the AI industry. In particular, we could further explore the factors influencing the AI index and analyze the characteristics of the AI industry in deeper detail. This would enable the construction of an accurate explanatory variables-based forecasting framework, which in turn could help investors grasp investment opportunities in the AI industry.

## Data Availability

The data can be obtained upon request.

## Abbreviations

AIRO: NASDAQ CTA artificial intelligence and robotics
EEMD: Ensemble empirical mode decomposition
ICSS: Iterative cumulative sum of squares
PSO-LSSVM: Least-square support vector machine approach with the particle swarm optimization
GARCH: Generalized autoregressive conditional heteroskedasticity
AI: Artificial intelligence
WHO: World Health Organization
NQROBO: Nasdaq CTA Artificial Intelligence and Robotics
ROBO: Global Robotics and Automation
IBOTZ: Indxx Global X Robotics & Artificial Intelligence
IMFs: Intrinsic mode functions
SN: Social network technique
RBF: Radial basis function
MSE: Mean square error
MAE: Mean absolute error
MCS: Model confidence set
ADF: Augmented Dickey–Fuller
PP: Phillips–Perron
AIC: Akaike information criterion
CER: Certainty equivalent return
NYFSRAI: NYSE FactSet Global Robotics and Artificial Intelligence

## Footnotes

1 The GARCH-type models can consider the conditional heteroscedasticity of financial time series and can capture some other characteristics, such as time-variation, volatility clustering (Mohammadi and Su 2010; Salisu and Fasanya 2013), while the AIRO index return series usually exhibit relevant characteristics.

2 We use the mean combination approach to obtain the corresponding forecasts. This is because some research points out that the simple mean forecast combination cannot be outperformed by other complicated forecast combination methods (Rapach et al. 2010; Claeskens et al. 2016).

3 Further information on the AIRO index is available at https://indexes.nasdaqomx.com/Index/Overview/NQROBO.
